# The Role of Comparisons in Judgments of Loneliness

**DOI:** 10.3389/fpsyg.2021.498305

**Published:** 2021-03-24

**Authors:** Andrew J. Arnold, Heather Barry Kappes, Eric Klinenberg, Piotr Winkielman

**Affiliations:** ^1^Department of Psychology, University of California, San Diego, San Diego, CA, United States; ^2^London School of Economics and Political Science, London, United Kingdom; ^3^Department of Management, New York University, New York, NY, United States; ^4^Department of Psychology, SWPS University of Social Sciences and Humanities, Warsaw, Poland

**Keywords:** comparisons, social comparison, loneliness, emotion, well-being, contrasts

## Abstract

Loneliness—perceived social isolation—is defined as a discrepancy between existing social relationships and desired quality of relationships. Whereas most research has focused on existing relationships, we consider the standards against which people compare them. Participants who made downward social or temporal comparisons that depicted their contact with others as better (compared to other people’s contact or compared to the past) reported less loneliness than participants who made upward comparisons that depicted their contact with others as worse (Study 1–3). Extending these causal results, in a survey of British adults, upward social comparisons predicted current loneliness, even when controlling for loneliness at a previous point in time (Study 4). Finally, content analyses of interviews with American adults who lived alone showed that social and temporal comparisons about contact with others were both prevalent and linked to expressed loneliness (Study 5). These findings contribute to understanding the social cognition of loneliness, extend the effects of comparisons about social connection to the important public health problem of loneliness, and provide a novel tool for acutely manipulating loneliness.

## Introduction

Loneliness, the emotional distress stemming from social connections that are perceived to be inadequate (Cacioppo and Patrick, 2008), is generally defined in terms of a discrepancy between perception of existing relationships and the idiosyncratic standards desired for those relationships. Nevertheless, most research on loneliness has focused on existing relationships, and surprisingly little attention has been given to the standards against which people compare them. The present line of research addresses this gap by examining how differences in comparison standards influence loneliness.

Loneliness can stem from dissatisfaction with the quantity *or* quality of relationships. For instance, loneliness is referred to as “a situation experienced by the individual as one where there is an unpleasant or inadmissible lack of (quality of) certain relationships. This includes situations in which the number of existing relationships is smaller than is considered desirable or admissible, as well as situations where the intimacy one wishes for has not been realized” (de Jong-Gierveld, 1987, p. 120). Although an objectively low quantity (few hours in the week spent with others) or quality (lack of close supportive friends) of contact with others is a risk factor for loneliness, the causal direction of this relation is unclear (Klinenberg, 2012), and a large body of research has shown that objective social contact and subjective loneliness are distinct constructs (e.g., Cutrona, 1982; Peplau and Perlman, 1982; Russell, 1996; Pressman et al., 2005; Dykstra and Fokkema, 2007).

Objective social contact and subjective loneliness are imperfectly related because of differences in the way people think about their contact with others—that is, because of intervening social cognition (Cacioppo and Patrick, 2008). Social cognition, therefore, is a promising route for understanding and influencing loneliness. There are three options for people who feel, or are at risk of feeling, lonely: increase the achieved level (quantity or quality) of social contact, decrease the desired level of social contact, or reduce the importance of the gap between the two (Perlman and Peplau, 1981). The latter two options, which are cognitive rather than behavioral strategies, appear to be both ubiquitous and potentially effective. Older adults indicated they would recommend to other lonely adults coping strategies that lower expectations about, or the importance of, social contact (Schoenmakers et al., 2012). Related research has shown that manipulating cognition, such as the salience of social connections, changes how people respond to social exclusion (Twenge et al., 2007). A meta-analysis of attempts to reduce loneliness found that the most successful interventions tested with randomized controlled trials (RCTs) were those that targeted maladaptive social cognition rather than actual social contact (Masi et al., 2011). However, these interventions were generally weeks- or months-long individual or group cognitive behavioral therapy sessions, in which many aspects of cognition were addressed (e.g., jealousy, communication, stress), so they do not clearly identify effects on loneliness of adjusting the desired level of social contact.

One influence on people’s *desired* levels of social contact is likely to be the perceived contact achieved by similar others: that is, social comparisons (Hyman, 1942; Festinger, 1954; Wills, 1981; Suls et al., 2002; Mussweiler, 2003). People are uncertain about their abilities and opinions, and reduce uncertainty by comparing themselves to others; these others provide a standard against which one’s own qualities—like intelligence or athleticism—may be evaluated (Festinger, 1954). People can be uncertain about loneliness too (e.g., Perlman and Peplau, 1981), so others’ quality and quantity of social contact may provide a standard against which one’s own social contact can be measured. Indeed, previous work on loneliness alludes to an effect of such comparisons. Dykstra et al. (2005) pointed to: “…the possible role of *social comparison* processes (Festinger, 1954) in late life loneliness. Older adults might be less lonely because they feel their social circumstances compare favorably in terms of earlier expectations or relative to peers” (p. 728). However, we are aware of little work that has directly tested the role of comparisons in loneliness.

Just as people feel worse about themselves in the presence of a highly competent other, and better about themselves in the presence of an incompetent other (Morse and Gergen, 1970), people should feel more lonely when comparing themselves to an individual with a better quality or quantity of social contact (upward comparison, Suls et al., 2002), and less lonely when comparing to an individual whose social contact is worse than their own (downward comparison). Indeed, Schoenmakers et al. (2012) describe a form of coping with loneliness that involves lowering expectations, which “…can be done by, for example, not expecting one’s children to visit as often, realizing that breaking down barriers to improve relationships is too costly, **or comparing oneself with someone who is worse off**.” (emphasis added; p. 354).

Similar others are not the only potential reference point for a comparison standard—oneself at other points in time also provides such a standard (Wilson and Ross, 2000). People feel better about themselves when they believe they have improved over time, and worse if they believe they have declined. If people evaluate loneliness using temporal comparisons of the present self to a past self, they should feel lonelier when comparing the present to a past with a better quality or quantity of social contact (upward comparison), and less lonely when comparing to a time in the past when social contact was worse (downward comparison). As with social comparisons, there is some evidence that people make temporal comparisons about their contact with others (Suls, 1986). In a longitudinal study of new students at college, loneliness was predicted by satisfaction with one’s social relationships, which in turn was related to comparisons with previous relationships as well as comparisons with one’s peers (Cutrona, 1982).

In sum, people should feel less lonely when they recognize their achieved (present) quantity or quality of social contact as surpassing a comparison standard, and lonelier when they see it as falling short of a comparison standard, whether these standards are social or temporal. Note that comparisons can focus on how the target and the self are similar as well as on how they differ (Mussweiler, 2003). However, because we consider comparisons in which one party is *better* and one is *worse*, our examination is confined to the comparisons that identify dissimilarities, referred to as contrasts. We first tested the effect of contrasts with three experiments in which people were instructed to make downward or upward social or temporal contrasts, and their feelings of loneliness were measured (Studies 1, 2, and 3). We then used a large-scale secondary survey dataset to see how contrasts were linked to loneliness over time (Study 4). Finally, we content-analyzed a sample of interviews with American adults living alone, to observe whether people spontaneously made social and temporal contrasts when they talked about their contact with other people, and whether these contrasts were linked to their feelings of loneliness (Study 5). We report how we determined our sample sizes, all data exclusions (if any), all manipulations, and all measures administered in each of the studies.

## Study 1

We hypothesized that people would feel less lonely when they made downward social or temporal contrasts, and more lonely when they made upward social or temporal contrasts. We had no reason to expect that one type of contrast (social vs. temporal) would be more effective, but we left this as an empirical question. We randomly assigned participants to make downward or upward social or temporal contrasts—or in a control condition, not to make any contrasts—before measuring their current feeling of loneliness.

Loneliness is most often measured using the 20-item revised UCLA Loneliness Scale (Russell, 1996), which we administered. However, the UCLA scale addresses feelings in general over an extended period of time: respondents indicate “how often” (*never, rarely, sometimes*, or *always*) they feel left out, isolated, shy, etc. If participants average their responses over an extended period of time, combining how they feel immediately after the manipulation with how they remember feeling in the recent past, then this scale provides a less-than-ideal tool for identifying an effect of the contrasts manipulation. Moreover, some UCLA scale items refer to commonalities with others (e.g., “My interests and ideas are not shared by those around me”) which might be affected by contrasts between one’s present and an alternative without necessarily tapping the emotional experience of loneliness. Accordingly, we also measured loneliness by simply asking participants how true it was that “right now, I feel lonely.”

### Methods

#### Participants and Design

Two hundred fifty-five individuals recruited *via* MTurk^1^,^2^ completed the survey materials in return for a $0.48 payment. We concluded data collection when reaching the pre-determined sample size of 50 per condition, which a G^∗^Power analysis (Faul et al., 2007) shows has 95% power to detect an effect size of *f* = 0.275 in a 5-group ANCOVA with two covariates. Four people were excluded from analysis for not writing as directed in response to the manipulation, as discussed in more detail in the Manipulation Check section below. The final sample of 251 included 127 men, 123 women, and one who identified as “agender,” ages 18–70 (*M* = 37 years, SD = 12.59). The experiment used a 2 (contrast direction: downward, upward) × 2 (contrast type: social, temporal) between-subjects design with an additional no-contrasts control condition.

#### Materials and Procedure

Participants were randomly assigned to one of the five experimental conditions. In the social contrast conditions, they read instructions that elicited contrasts between their own and others’ living situations:

First, we are interested in how your present living situation (who you live with, where you live, how you live) *compares to other people’s living situations*. In the space below, please briefly describe two ways that your present living situation is [better/worse] than other people’s living situations.

The text in brackets differed depending on whether participants were assigned to make downward or upward contrasts. Participants in the downward contrasts condition were asked to identify ways their own living situation was better, and those in the upward contrasts condition were asked to identify ways their own living situation was worse. We used parallel instructions in the temporal contrast conditions to elicit contrasts between present and past living situations, except that we removed the text that appears in italics above, and instead asked participants to describe how their present living situation: “…*compares to your living situations in the past*.” The fifth group of participants, assigned to a control condition, were not asked to make any contrasts and proceeded immediately to the measures below.

Thereafter, participants were asked: “Right now, how true is this statement of you? ‘I feel lonely.”’ The 7-point response scale had the options *extremely untrue* (1), *moderately untrue* (2), *somewhat untrue* (3), *neither true nor untrue* (4), *somewhat true* (5), *moderately true* (6), and *extremely true* (7). They then completed the 20-item Revised UCLA Loneliness Scale (Russell, 1996), which asks respondents to indicate “how often you feel the way described in each of the following statements,” where options are *never* (1), *rarely* (2), *sometimes* (3), and *often* (4). We computed the sum of the 20 items for each participant after reverse-coding the appropriate items (α = 0.96). Participants also reported their gender, age, relationship status, and living situation (live alone or live with other people), and provided any comments they wished to, before being presented a code with which to obtain payment *via* MTurk.

### Results and Discussion

#### Manipulation Check

A member of the research team read all responses, and four respondents that did not follow instructions (i.e., did not describe elements of their present living situation) were excluded from analysis.

Initial examination of the responses showed that in many cases, it was not possible to distinguish between social and temporal contrasts. For example, a participant wrote: “I have personal space that no one else can enter.” This is clearly a downward contrast but it’s not clear whether the contrast is to other people who do not have personal space, or to a time in the past when the participant did not have personal space. Other examples where direction can be inferred but social vs. temporal cannot are: “There is no fighting” and “I don’t get to see my friends very often.” While reading, the researcher also coded whether or not each respondent mentioned other people. This coding was used in follow-up exploratory analyses described below.

#### Preliminary Analyses: Demographic Characteristics and Loneliness

Although only a minority of participants (35; 14%) lived alone, they reported more loneliness than those who lived with others, both in terms of current feelings (*M*_*Alone*_ = 4.23, SD = 1.88 vs. *M*_*Others*_ = 2.94, SD = 1.84) and on the UCLA scale (*M*_*Alone*_ = 49.09, SD = 12.73 vs. *M*_*Others*_ = 40.69, SD = 13.47), *t*s(249) > 3.44, *p*s ≤ 0.001. Men and women did not differ in loneliness, *t*s(248) < 0.92, *p*s > 0.35, but age was related to loneliness, such that older participants reported less momentary loneliness, *r*(249) = −0.12, *p* = 0.008, and marginally less loneliness on the UCLA scale, *r*(249) = −0.12, *p* = 0.055. With participants ranging in age from 18 to 70, these negative correlations are in line with research finding that loneliness is higher in late adolescence and young adults than in middle-aged adults [review by Qualter et al. (2015)]. Importantly, randomization was effective; the portion of participants living alone vs. with others did not differ across the experimental conditions, χ^2^(4) = 6.87, *p* = 0.14, nor did participant age differ across condition, *F*(4, 246) = 1.56, *p* = 0.19. To increase the power to detect an effect of the contrast manipulations over and above the role of these other factors, we adjusted for living status and age in subsequent analyses.

#### Momentary Loneliness (Single-Item Measure)

**FIGURE 1:**
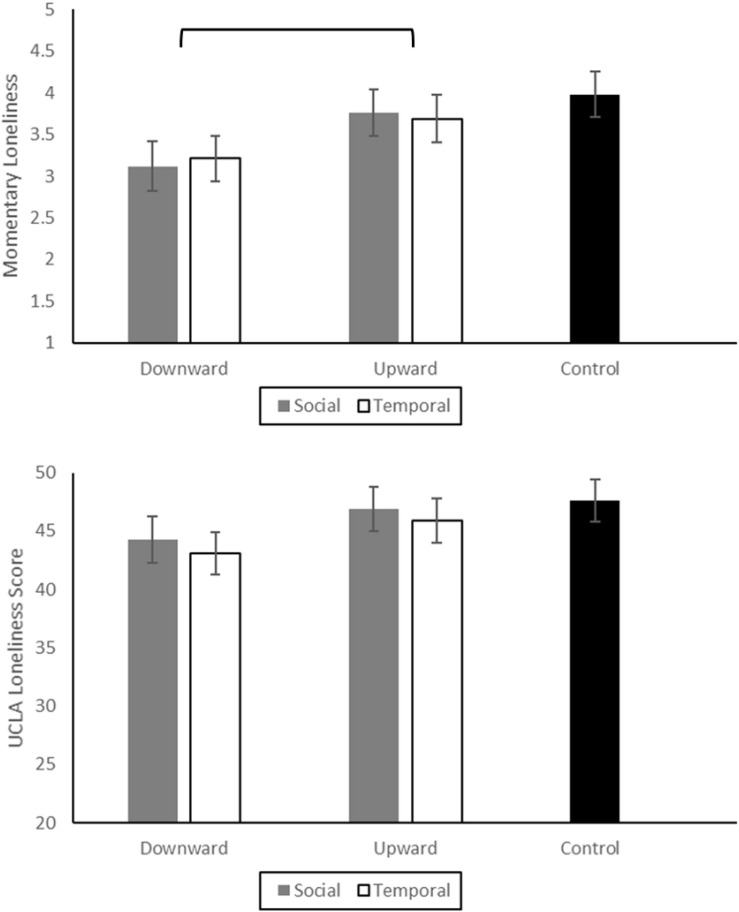
Adjusted marginal means for each condition for Study 1. Since the control condition included no contrasts, we present it separately in black. Momentary loneliness is a single item 7-point response scale and the UCLA scale has 20 items with a 4-point response scale. These values are adjusted for age and living status (alone, with others). Error bars are standard error and brackets indicate significant differences at the *p* < 0.05 level.

Because the design was not fully factorial (2 × 2 plus a control condition), we began by simply assessing differences across the five conditions, using an ANCOVA with condition as a between-subjects factor and age and living status (alone or with others) as covariates. When the single-item measure of current loneliness was the dependent variable, the effect of condition was not significant at the *p* < 0.05 level, *F*(4, 244) = 2.14, *p* = 0.07. Nevertheless, given the preliminary and thus somewhat exploratory nature of this initial study, we conducted a series of contrasts to answer specific research questions. We calculated adjusted marginal means for both momentary loneliness (single-item) and UCLA loneliness by condition. These group means, adjusted for living status and age, are depicted in Figure 1.

First, we compared downward to upward contrasts, collapsing across the social vs. temporal conditions. As predicted, downward vs. upward contrasts produced relatively lower vs. higher loneliness, *F*(1, 194) = 4.85, *p* = 0.029, η^2^_*partial*_ = 0.023. Next, we tested whether downward contrasts reduced loneliness, and whether upward contrasts increased loneliness, compared to the control condition. Downward contrasts did reduce loneliness, *F*(1, 151) = 7.49, *p* = 0.007, η^2^_*partial*_ = 0.047, but upward contrasts did not affect loneliness compared to the control condition, *F*(1, 145) = 0.67, *p* > 0.25. Finally, we tested whether social vs. temporal contrasts had different effects on loneliness. They did not; participants who made downward contrasts were similarly lonely if these contrasts were social or temporal, *F*(1, 92) = 0.795, *p* > 0.25, and participants who made upward contrasts were also similarly lonely whether their contrasts were social or temporal *F*(1, 98) = 0.01, *p* > 0.25. This was not surprising given that our examination of participants’ written responses to the contrast manipulations suggested that contents of social and temporal contrasts were largely indistinguishable.

As noted in the “Manipulation Check” section above, some participants’ contrasts referred to contact with others (e.g., I do/do not live with a loving partner) while some did not (e.g., I do/do not have spare money). It is conceivable that the latter types of issues still have downstream effects on contact—having no spare money might prevent one from spending time with friends or meeting new people, for instance. However, these types of contrasts do not unambiguously alter the comparison standard for determining a desired level of social contact, and so they might have weaker or no appreciable effects on loneliness (see Swann et al., 2007). As this was the first study and somewhat exploratory in nature, we wondered whether (social or temporal, downward or upward) contrasts focusing on contact with other people have stronger effects on subsequent loneliness. To examine this question, we divided participants in the contrast conditions into those who had generated one or two contrasts mentioning other people (*n* = 95) and those who had not generated any contrasts mentioning other people (*n* = 103), and repeated the analyses above separately for these two groups.

For participants whose contrasts mentioned other people (plus participants in the control condition), an ANCOVA with the five experimental conditions as a between-subjects factor and age and living status as covariates showed a significant effect of condition on feelings of loneliness, *F*(4, 141) = 2.823, *p* = 0.027, and η^2^_*partial*_ = 0.069. As in the full sample, downward vs. upward contrasts reduced loneliness, *F*(1, 91) = 6.808, *p* = 0.011, η^2^_*partial*_ = 0.065, and downward contrasts reduced loneliness compared to the control condition, *F*(1, 108) = 9.17, *p* = 0.002 η^2^_*partial*_ = 0.078, but upward contrasts did not affect loneliness compared to the control condition, *p* > 0.25.

For participants whose contrasts did *not* mention other people, the ANCOVA showed no effect of condition, *F*(4, 149) = 0.64, *p* > 0.25, η^2^_*partial*_ = 0.017, and so we did not conduct any follow up contrasts. Although these results must be interpreted with caution because participants were not randomly assigned to make comparisons about contact with others vs. comparisons about other aspects of the living situation, they suggest—as one would expect—that it is contrasts pertaining to contact with other people that appreciably affect loneliness, at least in a sample of this size. In other words, the effect of our contrast manipulation was only found for the 52% of the 198 participants in the contrast conditions who mentioned other people in their contrasts.

This finding is useful in speaking against an availability bias or mood-based explanation for the results. Participants who thought about how their house was comparatively bigger or income comparatively better should have felt happier, and had a heightened availability of mood-congruent thoughts, than participants who thought about how their house was smaller or income worse. However, these participants did not differ in the loneliness they reported, speaking against such mundane explanations for the manipulation’s effects.

#### UCLA Loneliness Scale

We followed the same series of steps to analyze UCLA Loneliness Scale scores. As with the single-item measure, an ANCOVA with the five experimental conditions as a between-subjects factor and age and living status (alone vs. with others) as covariates showed no significant effect of condition, *F*(4, 244) = 1.20, *p* > 0.250. The UCLA scores by condition mirror the pattern of self-reported current feelings of loneliness (see Figure 1), the differences were just smaller. However, when we tested effects on UCLA scores for participants whose contrasts mentioned other people (plus participants in the control condition), there was a significant effect of condition on feelings of loneliness, *F*(4, 141) = 3.48, *p* = 0.01, η^2^_*partial*_ = 0.084. Just as with momentary feelings of loneliness, in this portion of the sample, downward vs. upward contrasts produced relatively lower vs. higher loneliness, *F*(1, 143) = 3.17, *p* = 0.002, η^2^_*partial*_ = 0.051, and downward contrasts reduced loneliness compared to the control condition, *F*(1, 108) = 4.93, *p* = 0.028, η^2^_*partial*_ = 0.044, but upward contrasts did not affect loneliness compared to the control condition, *F*(1, 85) = 0.44, *p* > 0.25.

These results represent initial support for the idea that loneliness is influenced by differences in the standard to which people compare their present achieved social contact. Identifying how achieved contact with others was better than a comparison target reduced loneliness compared to identifying how achieved contact was worse than a comparison target. These results are consistent with the idea that momentary social cognition—for instance, the relationships and standards presently on one’s mind—can exert powerful effects on judgment. Here these results extended to answers on the UCLA loneliness scale, a trait measure—suggesting that even relatively fleeting social cognition can influence the way that people retrospect on and report their experiences over the recent past.

Secondary to the difference between participants who made downward vs. upward contrasts, we saw that downward contrasts reduced loneliness compared to a no-contrasts control condition, suggesting that such contrasts might be an effective intervention against loneliness. Although this recommendation is consistent with the finding that the most successful RCT-tested interventions against loneliness target social cognition (Masi et al., 2011), one must consider that reducing loneliness compared to a control condition depends on the average level of loneliness for control participants and perhaps on their existing social cognition; we do not know what kinds of contrasts, if any, control condition participants mentally make when they evaluate and report on their loneliness. Since an intervention to reduce loneliness is likely to be most effective when developed using samples of individuals with high levels of loneliness, in our non-clinical samples we instead focused on replicating and understanding the relative effects of making downward vs. upward contrasts.

## Study 2

The aim of Study 2 was to replicate the effect on loneliness of downward vs. upward contrasts. In order to strengthen this effect, and in hopes of identifying it in the whole sample rather than a subsample (based on the content of the contrasts), we explicitly instructed all participants to make contrasts about contact with others. As in Study 1, however, they were free to consider the quantity or quality of contact, or both dimensions.

We further utilized a portion of the sample in Study 2 to test another question of interest: would the effects of the manipulation be sustained over time? We did not necessarily anticipate that they would be, since the effects of social cognition on judgment should dissipate when the cognition changes. However, it was conceivable that effects would linger temporarily; we conducted seven daily follow-ups with a sub-sample of participants to see if this was the case, and if so, how long the effects persisted.

### Method

#### Participants and Design

Six hundred and thirty-one individuals in the United States recruited *via* MTurk, who had not participated in Study 1, completed the baseline survey materials in return for a $1.00 payment; a subset received an additional payment of up to $2.00 for completing follow-up surveys. We used a target sample size of 150 per cell and omitted the no-contrast control condition. This change meant that data would be analyzed with a 2 (contrast direction: downward, upward) × 2 (contrast type: social, temporal) between-subjects ANOVA. With two covariates (as in Study 1) this sample size had 98% power to detect an effect of the size observed in Study 1 (Faul et al., 2007). Upon content analysis, 30 (4.7%) were excluded since they did not complete the contrasts as assigned. The final sample included 341 men, 259 women, and one person who identified gender as “FTM.” Respondents were ages 18–82 (*M* = 32 years, SD = 9.80). Participants were randomly assigned to one cell of the 2 (contrast direction: downward, upward) × 2 (contrast type: social, temporal) between-subjects design (*ns* per cell = 147–154).

#### Materials and Procedure

The initial survey was similar to the materials and procedure of Study 1. The contrast manipulations were modified such that participants were asked to make comparisons about contact with other people. We provided an example of the relevant comparison in order to make sure that the instructions were clear. All participants first read:

First, we are interested in how your present living situation (who you live with, where you live, how you live) compares to other people’s living situations, specifically in terms of contact with other people (who you interact with, how those interactions go).

Thereafter, they read text that differed by condition (the text in italics is the portion that differed). In the downward social contrast condition, instructions read:

For example, you might think that your living situation is *better* than other people’s *because you live with someone whose interests are compatible with your own, and many people don’t*. This is just an example; you should come up with your own answers. In the space below, please briefly describe two ways that your present living situation, in terms of contact with other people, is *better* than other people’s living situations.

In the upward social contrast condition, instructions read:

For example, you might think that your living situation is *worse* than other people’s *because many people live with someone whose interests are compatible with their own, and you don’t*. This is just an example; you should come up with your own answers. In the space below, please briefly describe two ways that your present living situation, in terms of contact with other people, is *worse* than other people’s living situations.

In the downward temporal contrast condition, instructions read:

For example, you might think that your living situation now is *better* than in the past *because now you live with people whose interests are more compatible with your own.* This is just an example; you should come up with your own answers. In the space below, please briefly describe two ways that your present living situation, in terms of contact with other people, is *better* than past living situations.

And finally, in the upward temporal contrast conditions instructions read:

For example, you might think that your living situation now is *worse* than in the past *because you used to live with people whose interests were more compatible with your own*. This is just an example; you should come up with your own answers. In the space below, please briefly describe two ways that your present living situation, in terms of contact with other people, is *worse* than past living situations.

After making the specified contrasts, participants completed the single-item measure of loneliness and the UCLA Loneliness scale. To camouflage the purpose of the study, we presented these items intermixed with five measures unrelated to loneliness. These measures asked participants about their liking for music, liking for reading, how much they had slept the previous night, how often in the past week they had eaten breakfast, and how often they had skipped meals; the latter two were taken from Hays et al. (1984), and shown to be unrelated to loneliness (Hays and DiMatteo, 1987). We then measured demographic information and gave the opportunity to comment as in Study 1.

For 7 days thereafter, we emailed a subsample of participants (*n* = 256) a link to complete a short survey that allowed us to test whether initial effects of the manipulation would be sustained. To camouflage the purpose of the study, for the first 6 days, participants were asked to name what they had eaten for lunch the previous day^3^ and to indicate how much they currently liked music and liked reading, as well as to answer the single-item question about loneliness. On the seventh day, participants were administered these items plus the UCLA Loneliness Scale and the two meal regularity items. They were asked how much they had enjoyed participating in the series of surveys and what they thought the study was testing. They were then provided with another opportunity to comment on the survey and thanked for participation.

### Results

#### Immediate Effects

As in Study 1, a sizable minority of participants (95; 15.8%) lived alone, and they reported more loneliness than those who lived with others, both in terms of current feelings (*M*_*Alone*_ = 3.65, SD = 1.86 vs. *M*_*Others*_ = 2.82, SD = 1.85) and on the UCLA scale (*M*_*Alone*_ = 44.34, SD = 14.25 vs. *M*_*Others*_ = 39.72, SD = 13.23), *t*s(581) > 3.07, *p*s < 0.01. As in Study 1, gender did not relate to either measure of loneliness, *F*s < 1, and older participants again reported less loneliness on the UCLA scale, *r*(599) = −0.09, *p* = 0.02. They also reported less momentary loneliness, though the relation was only marginally significant this time, *r*(599) = −0.07, *p* = 0.07. Just as in Study 1, therefore, we adjusted for living status and age when testing the effects of the contrast manipulations.^4^

**FIGURE 2:**
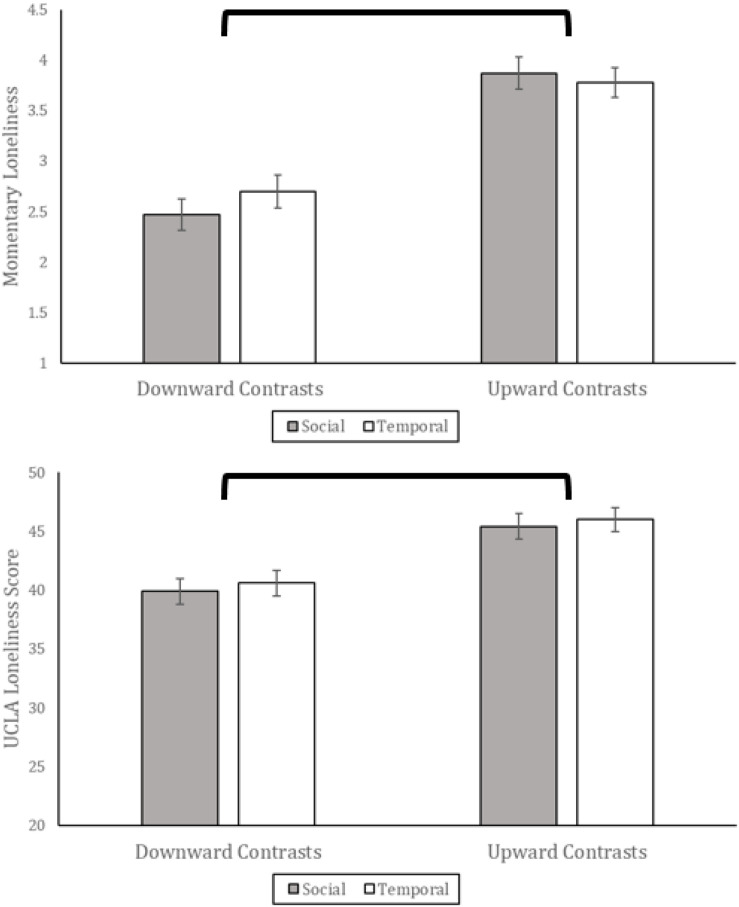
Adjusted marginal means for each condition for Study 2. Momentary loneliness is a single item 7-point response scale and the UCLA scale has 20 items with a 4-point response scale. These values are adjusted for age and living status (alone, with others). Error bars are standard error and brackets indicate significant differences at the *p* < 0.05 level.

We modified our analysis strategy from Study 1. Since there was no control condition we used a 2 (contrast direction: downward, upward) × 2 (contrast type: social, temporal) factorial ANOVA to test the effects of the contrast manipulations. In addition, we analyzed the two dependent variables (current feelings of loneliness and UCLA scale scores) simultaneously. The two measures of loneliness were strongly correlated, *r*(599) = 0.66, *p* < 0.001, although not so highly as to be collinear, satisfying the requirement for MANOVA (e.g., below 0.8; MANOVA Assumptions, 2020). A MANOVA with age and living status (alone, with others) as covariates showed a multivariate effect of contrast direction, *F*(2, 595) = 38.02, *p* < 0.001, η^2^_*partial*_ = 0.11, no multivariate effect of contrast type, *p* > 0.25, and no multivariate interaction effect of contrast direction by type, *p* > 0.25. Adjusted marginal means are presented in Figure 2. Whether social or temporal in nature, downward contrasts reduced loneliness compared to upward contrasts on the single-item measure of current feelings, *F*(1, 595) = 76.25, *p* < 0.001, η^2^_*partial*_ = 0.11, and on the UCLA scale, *F*(1, 595) = 31.60, *p* < 0.001, η^2^_*partial*_ = 0.06. These effects remained strong when omitting age and living status as covariates (*p*s < 0.001).

#### Sustained Effects

**FIGURE 3:**
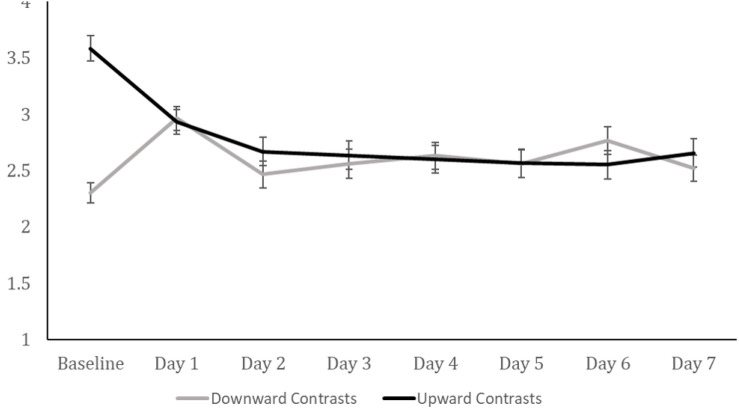
Average reported momentary loneliness at baseline, and over seven further days, following the first contrast made. Error bars are standard error and the only significant group difference based on direction of contrast was found at baseline.

Next, we tested whether differences in loneliness following the manipulation were sustained, for the set of participants who were contacted with innocuous daily follow-up surveys (*n* = 256). To do so we analyzed their daily reports of loneliness using Generalized Estimating Equations. This analysis has the advantage of including all participants who completed at least one follow-up survey, unlike a traditional repeated-measures analysis where only all the participants who completed all follow-ups would be analyzed. The predictors were baseline contrast direction (downward, upward), baseline contrast type (social, temporal), and day, plus all interaction effects. Again, living status and age were included as covariates. There was a significant effect of day, Wald χ^2^(1) = 21.99, *p* = 0.003, and a contrast direction by day interaction effect, Wald χ^2^(1) = 46.21, *p* < 0.001. Pairwise comparisons showed that although participants who made downward contrasts reported less loneliness than those who made upward contrasts immediately after the manipulation, *p* < 0.001, this difference was erased by the first follow-up survey, *p* > 0.25, and not detectable at subsequent follow-ups (see Figure 3). The lack of difference between conditions on Days 1–7 indicates that the effects of the manipulation do not persist over time, at least not to an extent observable in a sample of this size.

In Study 1, compared to the control condition, downward contrasts reduced loneliness, but upward contrasts did not significantly increase loneliness. One might therefore expect that the difference between downward and upward contrasts immediately after the manipulation (“Baseline”) is driven more by downward than upward contrasts; that loneliness in the upward contrasts condition is close to a theoretical control condition level. If this were the case, then we might also expect that loneliness on the follow-up Days 1–7 would be close to this level. Instead, Figure 3 highlights a relatively large reduction in loneliness in the days after making upward contrasts, and a relatively small increase in loneliness in the days after making downward contrasts. The picture painted by Figure 3 implies that each manipulation influenced loneliness (in opposite direction) relative to a hypothetical control condition, although we can only infer this given that there was no true control condition in this study.

As in Study 1, we hesitate to draw conclusions about one or the other condition driving the effect that we observed immediately after the manipulation, since it is likely to depend on participants’ initial levels of loneliness. We addressed this question in Study 3.

## Study 3

In Study 3, we used scores on the UCLA scale to divide participants into groups of low vs. high loneliness, before asking them to make downward or upward social contrasts about their contact with others. This served two goals. First, with content analysis we could test whether people who were high in loneliness were *able* to make downward contrasts about their contact with others, and whether people who were low in loneliness were able to make upward contrasts, when asked to do so. Our supplementary analyses in Study 1 found a strong effect of the manipulation, on both momentary (single-item) loneliness and the UCLA scale, among participants whose contrasts mentioned other people. One mundane explanation for this finding is an attrition bias: participants in the downward contrasts condition who were extremely lonely refrained from making contrasts about their contact with other people (and mentioned their income or the size of their house instead) because they were unable to make such downward contrasts. Finding that participants who are high in loneliness can in fact make downward contrasts about contact with others, and that participants low in loneliness can make upward contrasts about such contact, would speak against this explanation.

Second, we tested whether the manipulation was differentially impactful for people who were high or low in loneliness to start with. To identify a sufficient sample of participants relatively high in loneliness, we used a university student sample where loneliness was known to be rather widespread. Because doing so limited the possible sample size, we omitted the temporal contrasts conditions, reasoning that social contrasts might be more relevant to these relatively young participants. Peer comparisons are known to be ubiquitous for young adults like these (Gibbons and Buunk, 1999).

Finally, we administered a measure of interpersonal closeness in order to test the specificity of the manipulation and the extent to which it might be due to demand characteristics. Manipulating the way that participants see their own social contact as exceeding vs. falling short of a standard for such contact should affect loneliness (e.g., Schoenmakers et al., 2012), but not the closeness participants feel to a specific other person. Finding that the manipulation affects feelings of loneliness but not interpersonal closeness would argue against demand characteristics as the explanation for the effect of the contrasts manipulation.

### Methods

#### Participants and Design

Two hundred forty-one undergraduate students at University of California, San Diego participated in the experiment for partial class credit. The sample included 44 men and 197 women, ages 18–35 (*M* = 20.62, SD = 2.13). The experiment used a 2 (social contrast direction: downward, upward) × 2 (initial loneliness: low, high) between-subjects design. As in Study 1, we aimed for 50 participants per condition after excluding incorrect responses. Content analysis, which we used as a manipulation check and exclusion criteria in the first two experiments, played an additional role here: It allowed us to test whether participants high in loneliness were able to make downward contrasts. Exclusions are therefore described in more detail below.

#### Materials and Procedure

Participants first completed a survey including basic demographic information and the UCLA scale (Russell, 1996) as well as the Ten-Item Personality Inventory (Gosling et al., 2003) and Beck Depression Inventory (Beck et al., 1996); the latter are not analyzed here. Cacioppo and Patrick (2008) (p. 271) report that high loneliness is defined as summed UCLA scale scores of 44 or higher, so we created two groups, low (*n* = 111) vs. high (*n* = 130), based on the cut-off score of 44.

Participants were then randomly assigned to make either two downward or two upward social contrasts using the instructions from Study 2. Thereafter they used a 7-point scale (1 = *extremely untrue*, 7 = *extremely true*) to indicate how a series of randomly-ordered statements applied to them. The measures included the single-item question about momentary loneliness (“I feel lonely”) as in Studies 1 and 2, and filler items about liking for music and reading as in Study 2. We also added a single-item pictorial measure of interpersonal closeness, the Inclusion of Other in the Self scale (Aron et al., 1992). The scale depicts two circles representing “self” and “other” in seven degrees of overlap (depicted in online materials), which participants were asked to use to indicate the level of perceived closeness with their “closest friend.”

Following these measures, we administered the Reading-the-Mind-in-the-Eyes test (Baron-Cohen et al., 2001) and the Empathy Quotient scale (Baron-Cohen and Wheelwright, 2004). These assessments addressed secondary hypotheses, and are not analyzed here. All test materials are posted at^5^.

### Results and Discussion

Of 241 respondents, 70 (29%) did not make both of the contrasts they were asked to; in other words, they did not provide two contrasts that involved mention of other people, as instructed, and similar to our past cited studies, they were excluded. They were roughly evenly distributed across the downward (*n* = 31, 25.2%) and upward (*n* = 39, 33.1%) contrast conditions, χ^2^(1) = 1.80, *p* = 0.18. A binary logistic regression analysis indicated that participants low rather than high in initial loneliness were marginally less likely to complete the manipulation as instructed, *b* = 0.53, Wald χ^2^(1) = 3.45, *p* = 0.063; the odds of failing to complete the two instructed contrasts were 1.71 times higher for participants low in loneliness. However, there was no interaction effect between initial loneliness group and contrast condition, *b* = 0.28, Wald χ^2^(1) = 0.95, *p* > 0.25, indicating that the heightened tendency of participants low in loneliness to not make the instructed social contrasts was equally true whether they were instructed to make downward or upward contrasts. This finding strengthens the conclusions drawn from the supplementary results of Study 1 by speaking against an attrition bias driving those results.

Next, we tested the effect of the manipulation on the 172 participants who made the two contrasts as instructed, constituting in this case a check that the experimental manipulation was completed. As in Studies 1–2, men and women did not differ in the dependent variable indicator of momentary loneliness, *t*(170) = 0.83, *p* > 0.25. In this sample, a very small number of participants (*n* = 6, 3%) lived alone; they did not differ in present loneliness from those who lived with others, *t*(170) = 0.16, *p* > 0.25. Age was also unrelated to present loneliness in this sample, *r*(170) = −0.08, *p* > 0.25, unlike in Studies 1–2, probably because of the small age range of participants in Study 3. Therefore, we did not include age or living status as covariates in the analyses below.

**FIGURE 4:**
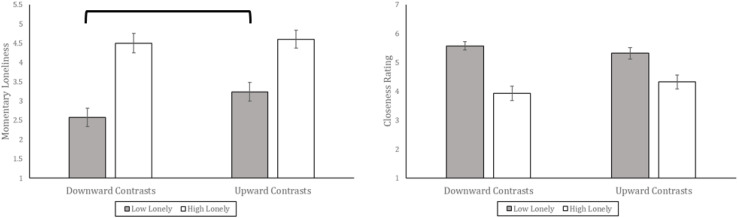
Momentary loneliness (single-item measure) and closeness to one’s closest friend as a function of downward vs. upward contrasts and initial loneliness (UCLA Loneliness scale score). These are group means and the error bars are standard error. The bracket indicates a finding within the Low Lonely group of contrast direction affecting loneliness at *p* = 0.05.

Momentary loneliness and interpersonal closeness were correlated, *r*(170) = −0.30, *p* < 0.001, so we next tested whether the effects of the manipulation would be specific to loneliness (rather than closeness), and whether these effects would depend on initial loneliness. To do so we conducted a repeated-measures ANOVA with on the scores, by adding measure (momentary loneliness or closeness) as a within-subjects predictor, along with the between-subjects predictors of loneliness group (low or high) and contrast condition (downward or upward). This analysis showed a marginally significant 3-way interaction effect of measure by contrast condition by initial loneliness, *F*(1, 168) = 3.57, *p* = 0.06, η^2^_*partial*_ = 0.021 (see Figure 4).

To clarify this interaction we conducted between-group *t*-tests based on condition, separately on groups of “low” or “high” initial loneliness from the UCLA scale. For participants initially low in loneliness, downward contrasts resulted in marginally lower momentary loneliness than upward contrasts, *t*(81) = −1.97, *p* = 0.053. For participants initially high in loneliness on the UCLA scale, the contrasts manipulation had no effect on momentary loneliness, *p* = 0.7610. The contrasts manipulation did not appreciably affect perceived closeness to one’s closest friend, for participants initially low in loneliness, or initially high in loneliness, *p*s > 0.250. The specificity of the manipulation’s effect—influencing loneliness but not interpersonal closeness—speaks against demand characteristics as an explanation.

In light of the effects of downward vs. upward contrasts seen in Studies 1 and 2, in samples where loneliness was rather low on average, it is probably unsurprising that the manipulation produced differences in loneliness for those students who were not highly lonely to start with. Nevertheless, this finding has important implications for the design of interventions against loneliness; it suggests that modifications would have to be made in order to utilize contrasts to decrease such feelings among the highly lonely.

In sum, the contrasts manipulation affected participants who were already low, but not high, in loneliness. This could, importantly, reflect an aspect of highly lonely individuals being somewhat resistant to such a brief contrast manipulation. It is possible that those already high in loneliness may not be affected by such a transitory consideration—whether they take it seriously or not—just because they may have already resigned to the “lonely mind” (Cacioppo and Hawkley, 2009).

High (trait) lonely participants didn’t change in their (state) loneliness, so one interpretation would be that the effects are driven by the upward contrasts condition (i.e., upward contrasts increase loneliness). This interpretation would be in line with the relatively large decrease in loneliness in the days following an upward contrasts manipulation that we observed in Study 2 (Figure 1). We therefore wondered what happens over time if people continue to make upward contrasts—do they experience sustained increases in loneliness? This question was not amenable to an experimental design since it would imply making people lonely (and perhaps inducing the negative health consequences of these feelings) over time. Instead, we used a panel survey.

## Study 4

In Study 4, we analyzed data from a population-representative sample of older adults in the United Kingdom. The measures of contrasts available in this panel study refer to courtesy and respect in service-based interactions (i.e., at restaurants, stores, or hospitals). These contrasts in Study 4 are more specific—and, one would expect, less important—than contrasts generated by participants in Studies 2–3, so we expected their effects to be weaker. However, the large representative sample that was contacted repeatedly in this study not only allowed us to track (small) predictive effects of upward contrasts on loneliness over time, it also complemented the American MTurk workers and university students who participated in Studies 1–3 to facilitate conclusions about generalizability.

### Methods

#### Participants and Design

The English Longitudinal Study of Aging (ELSA) includes approximately 12,000 respondents recruited to provide a representative sample of the English population aged 50 and over. Further information about the sample and methodology is available at^6^. We analyzed data from Waves 4 (2008–2009; *n* = 11,050), 5 (2010–2011; *n* = 10,275), and 6 (2013–2014, *n* = 10,601).

#### Materials

The complete list of measures administered per wave is available at^7^. In order to test how upward contrasts relate to loneliness over time, we identified measures of both variables, as well as appropriate control variables, from the items administered.

##### Contrasts

At Wave 5 only, three items pertaining to upward social contrasts were presented in a section with the instructions: “In your day-to-day life, how often have any of the following things happened to you?” The first item asked whether “You are treated with less courtesy or respect than other people,” the second asked whether “You receive poorer service than other people at restaurants or stores,” and the third was “You receive poorer service or treatment than other people from doctors or hospitals.” For all items, the response options were *almost every day* (6), *at least once a week* (5), *a few times a month* (4), *a few times a year* (3), *less than once a year* (2), and *never* (1). There were 7,901 valid responses to the three items and their internal reliability was acceptable (α = 0.68), so we summed responses to these items as an indicator of the frequency of upward social contrasts (ranged from three to 18, *M* = 4.91, SD = 2.16).

##### Loneliness

At Waves 4, 5, and 6, two items in the ELSA survey measured loneliness. On the first, respondents indicated whether or not they had felt lonely much of the time during the past week (*no* = 0, *yes* = 1). The second item was: “How often do you feel lonely?” with response options *hardly ever or never* (1), *some of the time* (2), and *often* (3). At all three waves, responses to the two items were strongly correlated [Wave 4 *r*(7346) = 0.57, *p* < 0.001; Wave 5 *r*(7988) = 0.57, *p* < 0.001; Wave 6 *r*(7712) = 0.56, *p* < 0.001] and were summed to create a single indicator of loneliness (ranged from one to four at each Wave, *M*_*T4*_ = 1.48, SD = 0.83; *M*_*T5*_ = 1.49, SD = 0.83; *M*_*T6*_ = 1.49, SD = 0.83).

##### Control variables

Particularly in light of the way that upward social contrasts were measured in ELSA, it was important to establish that any link between contrasts and loneliness was not spuriously related to a third variable such as a negative worldview, or generalized negative affect. As the best available items to control for such a third variable, we used items intended to measure personality dimensions of neuroticism and agreeableness (Saucier, 1994). Cacioppo and Hawkley (2009) reported that the personality dimensions predictive of loneliness included high neuroticism and low agreeableness (see Marangoni and Ickes, 1989; Cacioppo et al., 2006). To measure neuroticism, ELSA participants were asked to indicate how well “Moody” and “Nervous” described them, and to measure agreeableness, participants were asked to indicate how well “Sympathetic,” “Warm,” and “Helpful” described them, using response options *a lot* (1), *some* (2), *a little* (3), and *not at all* (4). These items were administered at Wave 5 only. We calculated the mean of the respective items to obtain indicators of neuroticism (α = 0.47, ranged from one to four, *M* = 2.96, SD = 0.69) and agreeableness (α = 0.70, ranged from one to four, *M* = 1.47, SD = 0.49). The two variables were only weakly correlated, *r*(8847) = −0.04, *p* < 0.001.

### Results and Discussion

First, we tested the cross-sectional relation of contrasts to expressions of loneliness, using the Wave 5 data. Thus, we modeled loneliness using multiple regression with amount of upward contrasts as a continuous predictor (Model 1). As expected, more frequent upward contrasts predicted higher concurrent loneliness, standardized β = 0.14, *t*(7797) = 12.36, *p* < 0.001, adjusted *R*^2^ = 1.9%. This relationship remained significant when controlling for neuroticism and agreeableness in Model 2, β = 0.08, *t*(7722) = 7.28, *p* < 0.001, adjusted *R*^2^ = 7.1%. As in previous work (Marangoni and Ickes, 1989; Cacioppo et al., 2006), in this multivariate analysis lower neuroticism predicted higher loneliness, β = −0.23, *t*(7722) = 20.31, *p* < 0.001, and lower agreeableness predicted higher loneliness, β = 0.05, *t*(7722) = 4.59, *p* < 0.001. Controlling for neuroticism and agreeableness helps to establish that the reason this measure of contrasts, which pertained to how one perceives treatment from others, relates to loneliness is not spuriously due to a negative way of seeing things. Results for both regression analyses are presented in Table 1.

**TABLE 1:** Regression analysis predicting loneliness at wave 5 from other wave 5 predictors.

	Adjusted R^2^	Predictors	β_*standardized*_	B	SE	*t* value	*P* value	VIF	AIC
Model 1	0.019	(Intercept)		1.218	0.023	53.12	<0.001		18,935.8
		Contrasts	0.139	0.053	0.004	12.36	<0.001		
Model 2	0.071	(Intercept)		2.006	0.056	35.63	<0.001		18,316.9
		Neuroticism	–0.229	–0.274	0.013	–20.31	<0.001	1.06	
		Agreeableness	0.051	0.086	0.019	4.59	<0.001	1.01	
		Contrasts	0.082	0.031	0.004	7.28	<0.001	1.06	
Model 3	0.433	(Intercept)		0.782	0.051	15.53	<0.001		12,290.1
		Loneliness at Wave 4	0.619	0.611	0.009	65.13	<0.001	1.07	
		Neuroticism	–0.102	–0.12	0.011	–10.48	<0.001	1.11	
		Agreeableness	0.032	0.054	0.015	3.5	<0.001	1.01	
		Contrasts	0.031	0.012	0.004	3.24	0.001	1.07	

Next, we tested the relationship between contrasts and loneliness over time. Loneliness was relatively stable over time (Wave 4 loneliness with Wave 5 loneliness *r*(6902) = 0.65, *p* < 0.001; Wave 5 loneliness with Wave 6 loneliness *r*(7269) = 0.68, *p* < 0.001), and sample sizes were slightly reduced by excluding participants who were missing responses at some waves. Nevertheless, contrasts at Wave 5 predicted loneliness at Wave 5 even when controlling for loneliness at Wave 4 along with controlling for neuroticism and agreeableness in Model 3, β = 0.03, *t*(6687) = 0.03, *p* = 0.001. Thus, in this population-representative sample of older adults in the United Kingdom, a small but reliable amount of the variance in loneliness was associated with upward social contrasts.

However, controlling for loneliness at Wave 5, contrasts did not predict loneliness at Wave 6, β = 0.014, *t*(7107) = 1.56, *p* = 0.12.^8^ In line with the theorizing above and results of the daily follow-up in Study 2, this result may speak to the importance of examining concurrent social cognition to understand loneliness. That is, contrasts are associated with loneliness at the same point in time, not in the future. If people make different contrasts (i.e., they change the way they think about their social contact), then loneliness should change.

Study 4 is valuable in showing a relationship between contrasts and changes in loneliness, which is not accounted for by personality indicators of a negative outlook on life, and which extends the earlier samples in age, culture, and representativeness. This relationship is particularly striking in light of the measure of contrasts, which by tapping courtesy and respect in service interactions, refers to contrasts that are more specific and probably less important than those identified in the experimental manipulations. In spite of their specificity and likely low importance, these contrasts explained variance in loneliness concurrently as well as from the past to the present. One limitation of the experiments (Study 1–3) that is not addressed in the survey design of Study 4, however, is whether people *spontaneously* make social and temporal contrasts when thinking about their contact with other people. We used content analysis in Study 5 to gain insight into this issue—how prevalent are such contrasts in conversations about daily life, what do they look like, and are they linked to expressions of loneliness.

## Study 5

In the course of research about the experience of solo living, Klinenberg (2012) interviewed middle-aged middle-class adults and older adults who lived alone. These were long-form, semi-structured interviews utilizing open-ended questions around the topic of living alone. Since contrasts were not the research topic of interest, participants were not asked whether or how they compared their social contact to others or to the past; therefore, we content-analyzed the interview transcripts to look for the presence of spontaneous contrast statements. We also noted whether or not participants, who lived alone and therefore were likely to have objectively low social contact, described themselves as lonely. To avoid coder bias producing a link between the presence of contrasts and perceived loneliness in a transcript, we used a multi-step coding method.

### Participants and Design

There were 122 transcribed one-on-one interviews collected by Klinenberg (2012); see data collection details on p. 235–237) available for analysis. Interview subjects were adults who lived alone in major metropolitan areas of the United States, primarily four boroughs of New York City (Brooklyn, the Bronx, Manhattan, and Queens). Age and gender information, where available, is noted below.

### Procedure

First, a research assistant read the 122 interviews and noted the interviewee’s gender and age (if specified) as well as whether or not the interviewee was asked about loneliness. Twenty-five interviews that did not include this question were excluded from analysis. The remaining sample of 97 included 69 women and 28 men ages 33–97 (19 interviewees did not provide their ages). In this sample, 48 interviewees (49%) reported being lonely (i.e., said “yes” when asked if they were lonely), 39 (40%) reported not being lonely (i.e., said “no”), and 10 (10%) gave an unclear answer.

In the second step of coding, one of three research assistants read each of the 97 interviews and extracted each statement that they saw as pertaining to comparisons about one’s life or living situation. They extracted 689 statements formed of one or more contiguous sentences, of which 314 (46%) were classified as social contrasts, 270 (39%) as temporal contrasts, and 105 (15%) as unclear or neither of these.

In the third step, the 584 social and temporal contrast statements from the 97 interviews were sorted in a random order and the identity of the interviewee was concealed. These statements were then coded by two research assistants as downward contrasts in which the present was better than the comparison standard, upward contrasts in which the present was worse than the comparison standard, or unclear/can’t tell. After the first pass coding, the research assistants discussed approximately one-third of the cases on which they had disagreed, before re-coding the remaining disagreements. This method yielded high inter-coder agreement, Cohen’s Kappa = 0.72. Of the 106 remaining disagreements, 75 (71%) were resolved by a third coder, and 31 (29%) that could not be resolved were discarded from analysis. This coding procedure resulted in 553 contrast statements from interviews with 96 participants; frequencies by direction and type, along with examples, are summarized in Table 2.

**TABLE 2:** Contrast frequencies and examples by type and direction in Study 5.

	Contrast direction		
Contrast type	Downward	Upward	Unclear
Social	130 (23.5%) A lot of single women feel like failures or something and they get a man and they’re just like oh good I’ve made it you know? And they’ll marry a guy that almost, well not that they can’t stand, but that bugs them and even that they’ve ion respect for or whatever but they’ve already put a year or two of doling into it and he’s basically harmless audit’s like going back into the dating world it would be like having your teeth pulled out They can’t deal with that… And I just see a lot of that as being sort of false and not really my priority because of fear of not having someone or because my ego needs it or I need die validation.	73 (13.2%) Despite the way I live I am a very relational person and, to me, meaning comes from relationships so when there are not people there sometimes I think too much about…you get existential problems about living alone. What is this for? Who am I giving it to? Where is the love in my life? All these questions come to bear on you when you live alone in a different way. I say that to other people and they say that’s not true, when you live with other people you get the same questions They’re just not as insisting because there’s more distraction.	93 (16.8%)
Temporal	103 (18.6%) And being alone, really alone is a lot easier than being that alone that’s because of the coldness in a relationship. I would much rather live alone then deal with something like that again.	121 (21.9%) I liked sharing the minutia of daily life, I liked things that—now that I live alone, so much of my daily experience never gets reported. But living with someone else you tell silly crazy things that don’t matter in the big scope, but they make you feel more like a person when those little things register, so I liked that. I liked being able to plan in person whatever we were going to do	33 (6.0%)

### Results and Discussion

The first thing to note is that contrast statements were common in the interviews. Considering only those contrasts where the direction was clear, interviewees made an average of 1.35 downward social contrasts (SD = 1.69), 0.76 upward social contrasts (SD = 1.06), 1.07 downward temporal contrasts (SD = 1.39), and 1.26 upward temporal contrasts (SD = 1.15). Eighty percent of interviewees made at least one clear downward contrast, and eighty-seven percent made at least one clear upward contrast. In the subset of participants (*n* = 80) where age could be identified, older participants were less likely to have made a downward temporal contrast, *r*(78) = −0.25, *p* = 0.026.

How did contrasts in the interviews relate to expressions of loneliness? When we compared the three groups of participants, who were lonely, not lonely, and unclear in their response, there was no difference in the mean number of contrast statements of the various types, *F*s(2, 93) < 1.09, *p*s > 0.25. However, in a binary logistic regression analysis, the presence (vs. absence) of *downward* temporal and social contrasts together marginally predicted being lonely (vs. not being lonely), χ^2^(2) = 5.04, *p* = 0.08. The coefficients on the dummy variables representing the presence of downward social contrasts, *b* = −0.72, exp(b) = 0.49, and downward temporal contrasts, *b* = −0.67, exp(b) = 0.51, indicated that the probability of being lonely was lower for participants who made these contrasts. The presence (vs. absence) of upward temporal and social contrasts, on the other hand, was unrelated to loneliness (vs. not being lonely), χ^2^(2) = 0.05, *p* > 0.25.

Why might the predictive links to expressed loneliness be driven by the presence vs. absence of (downward) contrasts, rather than the number of contrasts of various types? Several factors are worth considering. Methodologically, extracting the comparative statements from their context—which has the benefit of preventing coder bias (i.e., coders were blind to participants’ loneliness when coding the direction of the contrasts)—has the side effect of leaving some statements unclear in direction. Presence vs. absence is thus measured with more precision than number. More interesting theoretically, it is possible that contrast statements that are particularly strong or meaningful to the participant—information that is impossible to discern from an interview transcript—might compensate for more, but weaker, contrasts of opposite direction (Swann et al., 2007). In sum, however, this content analysis suggests both the prevalence of spontaneous social and temporal contrasts about contact with others, and a link between those contrasts and loneliness.

## General Discussion

Loneliness stems from the perception that the present living situation has inadequate social connection (Cacioppo and Patrick, 2008). As with many perceptions, *inadequacy* here is determined by comparing the present to a criterion, such as social connection *apparently* achieved by others or in one’s own past. When the present living situation surpasses the criterion, people should feel less lonely than when the present living situation falls short of a criterion. In line with this speculation, the results from five studies suggest that downward contrasts, which depict the present quality and/or quantity of social contact as better than a given standard, produce lower loneliness than upward contrasts, which depict the present social contact as worse than a standard. These results contribute to an important gap in the literature on loneliness, which is generally defined in terms of a discrepancy between existing relationships and the standards desired for those relationships. Whereas previous research has largely focused on the existing relationships, the present studies show that the other component of the definition also plays an important, even causal, role.

The mixed methods of these studies contribute different strengths. The first three, with experimental designs, show a causal relation between contrasts and loneliness. Although this relation may well be bidirectional—lonely people probably have a tendency to see themselves as relatively worse off—very briefly induced downward vs. upward contrasts produced consistent differences in loneliness, demonstrating that in this direction the relation can be understood as causal. The large survey dataset analyzed in Study 4 indicated that upward social contrasts (even in specific and minor life domains) can explain variance in both concurrent loneliness and changes in loneliness over time, in a population-representative sample of older adults. And adding richness to the experimental and survey data, the content analyses in Study 5 suggest that temporal and social contrasts are a common ingredient in thoughts and conversations about daily life among individuals at risk of feeling lonely (i.e., solo dwellers).

The contributions of this research are both theoretical and practical. On the theoretical side, we show that loneliness is influenced by the standards against which people compare their social connections. This finding is fully in line with work that defines loneliness as a discrepancy between existing relationships and the standards desired for those relationships (e.g., Cacioppo and Patrick, 2008)— supporting it empirically complements the bulk of research that has focused on determinants in terms of relationships themselves rather than standards. It is also interesting theoretically to note that when social and temporal contrasts were both examined (Study 1, 2, and 5), they appeared to exert similar effects. Note that there may be groups for whom one or the other type of contrast comes more naturally or is more powerful (see e.g., Lyubomirsky and Ross, 1997). However, in our studies both content and effects of the two types of comparisons were largely indistinguishable. The present research also suggests that downward contrasts may decrease loneliness (Study 1 and 5) and upward contrasts may increase loneliness (Study 2, 3, and 4) compared to some reference value, but more research is needed on this point. We suspect the answer will depend at least in part on the level of loneliness and the style of thinking with which participants begin.

One practical implication concerns how to study downstream consequences of loneliness. Most investigations use correlational methodology. When experiments are utilized, they have relied on time- and labor-intensive methodologies like hypnosis to induce loneliness (Cacioppo et al., 2006). Future research can use the quick and inexpensive identification of contrasts to induce relatively high vs. low loneliness and study downstream consequences. Importantly, the effect of the contrast manipulation on momentary loneliness was mainly prevalent only when participants engaged in contrasts that mentioned other people.

A further practical implication concerns interventions against loneliness. Such interventions are often based on changing existing relationships—introducing participants to new people or helping them feel closer to those they know. Or, one might change the relationships that are salient; for instance, reminding people about their social connections, which enhances trust in others, reduces aggression in response to social exclusion (Twenge et al., 2007). The present research suggests that targeting the standards against which these relationships are evaluated is a fruitful avenue to explore, but more exploration is needed. Indeed, interventions targeting social cognition appear to be the most beneficial (Masi et al., 2011), but they often involve weeks- or months-long sessions of cognitive therapy. Future research might explore how to make the effects of contrasts identified here more powerful, perhaps by having participants make more than two contrasts, by inducing social and temporal contrasts at the same time (see Zell and Alicke, 2009), by building on temporal contrasts to help participants generate counterfactual statements about what they could have done differently and could do differently in the future (Epstude and Roese, 2008; Smallman and Roese, 2009), or by harnessing assimilation processes as well as contrasts. Then, incorporating contrasts into social cognition interventions might make those interventions more expedient as well as more effective.

The present studies focused on downward and upward comparisons in which people identify dissimilarities between their present living situation and a standard. One should note that making comparisons by identifying similarities, which leads to assimilation rather than contrast in judgment (Mussweiler, 2003; Bless and Schwarz, 2010), might produce effects opposite to those hypothesized and identified here. For example, people instructed to identify ways that their living situations were *similar to* someone else’s living situation might feel less lonely if that someone else had a high rather than low quality of social contact. Assimilation processes explain why merely seeing a well-off target (e.g., someone with extremely high-quality relationships) does not necessarily make observers feel lonely (Bless and Schwarz, 2010). Interventions against loneliness based on social comparisons might therefore induce both downward contrasts and upward assimilation.

In addition, future research might usefully extend the examination of comparison processes. For instance, one could examine lateral comparisons (i.e., no difference between self and other; no change between past and present), or comparisons to a possible future self. This latter type of comparison might occur spontaneously if people assimilate their circumstances to a downward social target and feel threatened that a possible future self could end up in the same situation as the worse-off other, or if people assimilate their circumstances to an upward social target and feel inspired that a possible future self could end up in the same situation as the better-off other (e.g., Strahan and Wilson, 2006). Future studies may also add mood measures taken (before and) after the manipulation, in order to rule out possible more generalized mechanisms of the manipulation’s impact on loneliness judgments. We also note that SES/income level was not incorporated in these studies, but as it shapes potential valuation of life conditions, it should be measured in future studies.

Finally, while the present paper focused on the role of social comparisons, it is important to remember that feelings of loneliness are determined by multiple sources. Classic work focused on aberrant processing of social stimuli that promote positive social interactions (Cacioppo and Hawkley, 2009). More recent theorizing highlights a possible role of interoceptive dysregulation, in which lonely individuals lose the ability to accurately “tune in” to one’s own internal, especially emotional, states and properly use them in social judgments (Arnold et al., 2019). Recent related research also highlights the deficits in spontaneous responding of lonely individuals to positive signals of social connection (Arnold and Winkielman, 2020). As such, future studies may explore the interaction of higher-order social comparison processes with these more basic mechanisms.

In sum, the primary contribution of this series of studies is the attention to the comparison standards that people use to evaluate their loneliness. Feelings of loneliness produce unmistakable emotional distress, often accompanied by a host of undesirable health consequences (Cacioppo and Patrick, 2008). As the present research highlights, these feelings depend not only on objective information about existing relationships, but also on the way that people think about those relationships and the standards against which people compare them.

## Data Availability Statement

All datasets generated for this study are included in the article/supplementary material.

## Ethics Statement

The studies involving human participants were reviewed and approved by UCSD IRB HRPP. The patients/participants provided their written informed consent to participate in this study.

## Author Contributions

HK conceived of the initial studies and drafted the first manuscript. AA contributed Study 3 and linkage with other studies, added substantial contributions and considerations, and including figures and analyses. EK and PW also provided crucial edits. All authors approved the current manuscript.

## Conflict of Interest

The authors declare that the research was conducted in the absence of any commercial or financial relationships that could be construed as a potential conflict of interest.

## Footnotes

1 Although concerns have been raised about the veracity of responses from MTurk participants, these concerns were much less prevalent when this study was conducted in early 2014, a time when research suggested that MTurk participants were similar to traditional samples in their responses to a variety of well-established research paradigms, and also similar to the general US population in their demographic characteristics (Buhrmester et al., 2011; Goodman et al., 2013; Paolacci and Chandler, 2014).

2 www.mturk.com

3 Another group of participants (*n* = 258) were asked to generate one downward contrast each day instead of reporting what they had eaten for lunch the previous day. Follow-up analyses from these participants are not presented here.

4 Unlike in Study 1, age and living status differed across condition. Note that power to find small effects statistically significant was much higher given the large sample size. Most importantly, the effects reported here are virtually unchanged if age and living status are not included as covariates; they are included for comparability with Study 1.

5 osf.io/6csyh

6 http://www.elsa-project.ac.uk/

7 http://www.ifs.org.uk/ELSA/documentation

8 Because contrasts were measured at only one Wave, this data is not amenable to more sophisticated investigation of causal relationships like a cross-lagged panel analysis. We did test the relationship between contrasts and changes in loneliness with a latent difference score model estimated in Mplus (version 7.11; Muthén and Muthén, 2012). This method produced conclusions identical to the regression analyses above: controlling for neuroticism and agreeableness, both of which predicted the change from Wave 4–5 but not the change from Wave 5–6, contrasts measured at Wave 5 predicted the change in loneliness from Wave 4–5, standardized coefficient 0.036, *p* < 0.001, but not the change in loneliness from Wave 5–6, standardized coefficient −0.018, *p* = 0.16.
